# Evaluation of EphA2 and EphB4 as Targets for Image-Guided Colorectal Cancer Surgery

**DOI:** 10.3390/ijms18020307

**Published:** 2017-02-03

**Authors:** Marieke A. Stammes, Hendrica A. J. M. Prevoo, Meyke C. Ter Horst, Stéphanie A. Groot, Cornelis J. H. Van de Velde, Alan B. Chan, Lioe-Fee de Geus-Oei, Peter J. K. Kuppen, Alexander L. Vahrmeijer, Elena B. Pasquale, Cornelis F. M. Sier

**Affiliations:** 1 Department of Radiology, Leiden University Medical Center, 2300 RC Leiden, The Netherlands; m.a.stammes@lumc.nl (M.A.S.); L.F.de_Geus-Oei@lumc.nl (L.-F.d.G.-O.); 2 Percuros B.V., 2333 CL Leiden, The Netherlands; achan@percuros.com; 3 Department of Surgery, Leiden University Medical Center, 2300 RC Leiden, The Netherlands; h.a.j.m.prevoo@lumc.nl (H.A.J.M.P.); m.c.terhorst@lumc.nl (M.C.T.H.); s.a.groot@lumc.nl (S.A.G.); c.j.h.vandevelde@lumc.nl (C.J.H.V.d.V.); p.j.k.kuppen@lumc.nl (P.J.K.K.); a.l.vahrmeijer@lumc.nl (A.L.V.); 4 Sanford Burnham Prebys Medical Discovery Institute, La Jolla, CA 92037, USA; elenap@sbpdiscovery.org; 5 Antibodies for Research Applications B.V., 2805 HT Gouda, The Netherlands

**Keywords:** tissue microarray, immunohistochemistry, cancer imaging, tyrosine kinase receptor, normal tissue, colon cancer

## Abstract

Targeted image-guided oncologic surgery (IGOS) relies on the recognition of cell surface-associated proteins, which should be abundantly present on tumor cells but preferably absent on cells in surrounding healthy tissue. The transmembrane receptor tyrosine kinase EphA2, a member of the A class of the Eph receptor family, has been reported to be highly overexpressed in several tumor types including breast, lung, brain, prostate, and colon cancer and is considered amongst the most promising cell membrane-associated tumor antigens by the NIH. Another member of the Eph receptor family belonging to the B class, EphB4, has also been found to be upregulated in multiple cancer types. In this study, EphA2 and EphB4 are evaluated as targets for IGOS of colorectal cancer by immunohistochemistry (IHC) using a tissue microarray (TMA) consisting of 168 pairs of tumor and normal tissue. The IHC sections were scored for staining intensity and percentage of cells stained. The results show a significantly enhanced staining intensity and more widespread distribution in tumor tissue compared with adjacent normal tissue for EphA2 as well as EphB4. Based on its more consistently higher score in colorectal tumor tissue compared to normal tissue, EphB4 appears to be a promising candidate for IGOS of colorectal cancer. In vitro experiments using antibodies on human colon cancer cells confirmed the possibility of EphB4 as target for imaging.

## 1. Introduction

During oncologic surgery, there is limited information available about the exact boundaries between tumor and healthy tissue. Visual inspection and palpation are often not enough, leading to incomplete resection of the tumor or substantial damage of healthy tissue [1]. Imaging of tumor tissue by targeted real-time near-infrared (NIR) fluorescence is a novel technique that can help the surgeon during an operation [1,2]. Besides the quality of the camera system, the effectiveness of this technique relies mainly on the choice of targeted tumor protein. Receptors and adhesion molecules upregulated on the surface of tumor cells are the best candidates for targeted cancer imaging, but the ideal protein for colorectal cancer imaging has not yet been identified [3]. Cell surface-associated proteins such as MUC1, EGFR, HER2, PSMA, and CEA are highly ranked on the National Cancer Institute (NCI) prioritization list of cancer antigens and are amongst the most pursued biomarkers for imaging [4]. We decided to evaluate one of the next candidates on the list, EphA2, a member of the Eph family of receptor tyrosine kinases that is preferentially expressed in tumor tissue compared to normal tissue and plays an important role in cancer malignancy [4,5]. Furthermore, we included the family member EphB4 because its overexpression has also been reported in various cancer types [6]. As a target for tumor imaging, a low grade of expression of both EphA2 and EphB4 in normal tissue is essential and has not been thoroughly investigated [5,6,7].

EphA2 and EphB4 are known to be upregulated, particularly in the early stages of colorectal cancer [5,8,9]. In those early stages, surgery without additional systemic therapy is the main treatment modality, which makes accurate recognition and removal of the tumor essential [10,11]. Whether a protein target like EphA2 or EphB4 is suitable for image-guided oncologic surgery (IGOS) is determined by its expression pattern in the tumor in comparison with the surrounding normal tissue. In this study, we evaluate the expression patterns of EphA2 and EphB4 by immunohistochemical (IHC) staining of a tissue microarray (TMA) consisting of pairs of tumor and normal colon tissue. Furthermore, the principle of using these Eph proteins for imaging is evaluated in vitro using various cancer cell lines.

## 2. Results

A total of 168 tumor/normal pairs were suitable for evaluation of both EphA2 and EphB4. The main characteristics of the patients and the tumors are presented in Table 1. In a preliminary evaluation on five sets of tumor and normal tissue sections, two different antibodies recognizing EphA2 and two different antibodies recognizing EphB4 showed similar staining patterns. Therefore, only one antibody recognizing each receptor (rabbit polyclonal 34–7400 for EphA2 and mouse monoclonal 37–1800 for EphB4) was selected for screening the TMA. All stained tissues were evaluated by two examiners and their scores showed a strong positive correlation (*R*^2^ = 0.86, *p* ≤ 0.001). Inter-observer agreement was obtained by re-evaluation of the respective sections.

**Figure 1:**
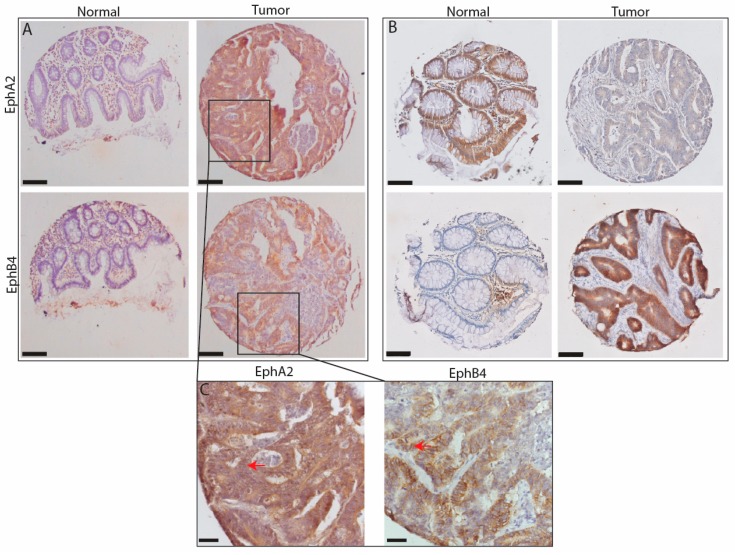
Two examples of staining patterns for EphA2 and EphB4 in sets of tumor and normal tissue from patients with colon cancer. (**A**) shows the most generally found pattern, with low expression of both proteins in normal tissue and abundant expression in tumor tissue; (**B**) shows an aberrant expression pattern (as indicated in Figure 2), with extremely high EphA2 expression in normal tissue and absence of staining in the corresponding tumor tissue. The scale bars represent 150 micrometers; (**C**) shows 40× enlargements of the sections indicated in A. Red arrows indicate membranous staining. The scale bar represents 30 micrometer.

Staining for EphA2 and EphB4 was generally present in epithelial cells throughout the whole tumor area consisting of 2 or 3 TMA cores (Figure 1). EphA2 staining was also widely detected in endothelial cells, whereas EphB4 staining was only occasionally found in endothelial cells. In general, tumor tissue showed more staining for both Eph receptors than the corresponding normal mucosa, but there were a few aberrant cases in which normal tissue showed more staining. Consequently, EphA2 and EphB4 expression did not significantly correlate (*R*^2^ = 0.08, *p* = 0.165, *n* = 336). Figure 1A,C shows a typical example of a Stage III patient with high EphA2 and EphB4 staining in the tumor and low staining in normal mucosa. Figure 1B presents a particular case in which normal tissue showed more EphA2 staining than the corresponding Stage III tumor. For EphA2, the mean score for the tumors (4.3 ± 1.8) was significantly higher (*p* < 0.001) compared with the corresponding normal tissue (3.3 ± 2.3). For EphB4, the difference between tumor and normal mucosa was even more pronounced, with scores of respectively 4.6 ± 1.6 versus 2.3 ± 1.9 (*p* < 0.001). There was no association between the clinical-pathological variables “tumor stage” or “differentiation” and the score for EphA2 or EphB4 staining.

**Figure 2:**
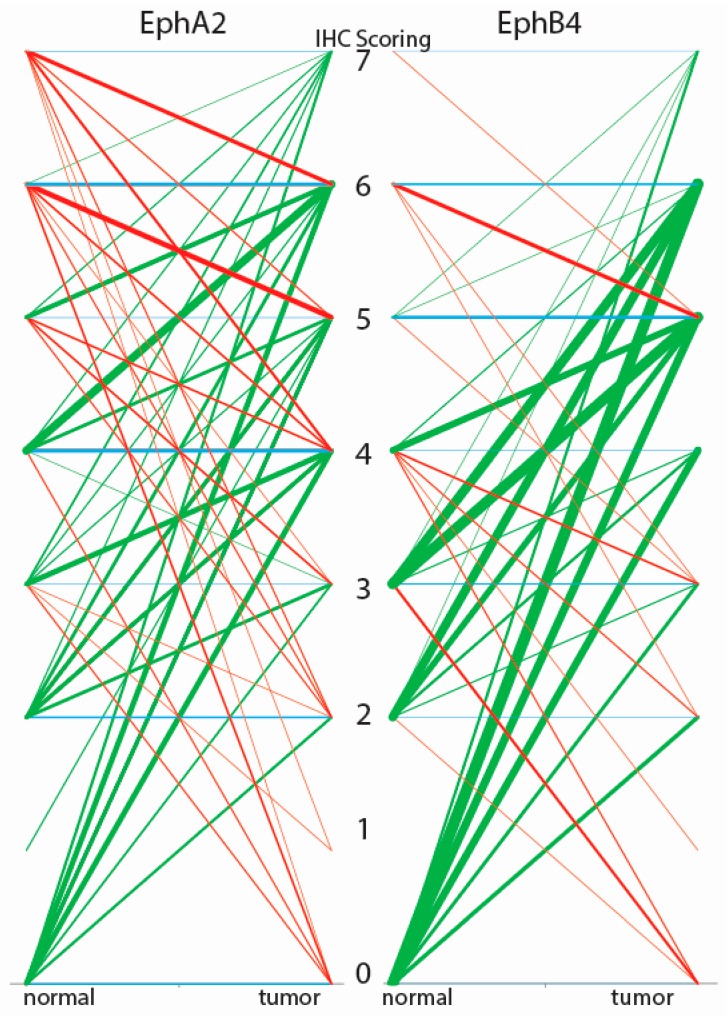
N/T scorings diagram for the tissue microarray (TMA) stained for EphA2 and EphB4. Green = higher score in tumor than normal tissue; Red = higher score in normal tissue than tumor tissue; Blue = no difference in score between normal and tumor tissue. The thickness of the line represents the number of patients, the thicker the line the more patients. The numbers 0–7 represent the IHC scoring values.

For imaging purposes, the over-expression of a protein target in tumors compared to adjacent normal tissue is more important than high tumor expression per se. Therefore, in the normal to tumor (N/T) scoring diagram in Figure 2, we show a graphical representation of the differences in EphA2 and EphB4 expression in normal versus tumor tissue for individual patients (Table S1). The IHC score of a normal tissue (left side) is connected by a line with the score of the corresponding tumor tissue (right side). Green lines indicate that the tumor score was higher than the score of the corresponding normal tissue, whereas red lines indicate the opposite. Blue lines indicate no difference between tumor and normal tissue. The thickness of the lines is proportional to the number of pairs with identical scores; the thicker the line, the more pairs there are. The diagram indicates that there is more variation in the N/T ratios for EphA2 than for EphB4. Although both Eph receptors show some unfavorable red lines, the majority of tissue sets (73% for EphA2 and 88% for EphB4) have green lines. Interestingly, the frequency of pairs with at least two score points difference between N and T is 46% for EphA2 versus 69% for EphB4. The frequency for a three-point difference becomes 30% for EphA2 versus 52% for EphB4, and for a four-point difference it becomes 23% for EphA2 versus 35% for EphB4.

**Figure 3:**
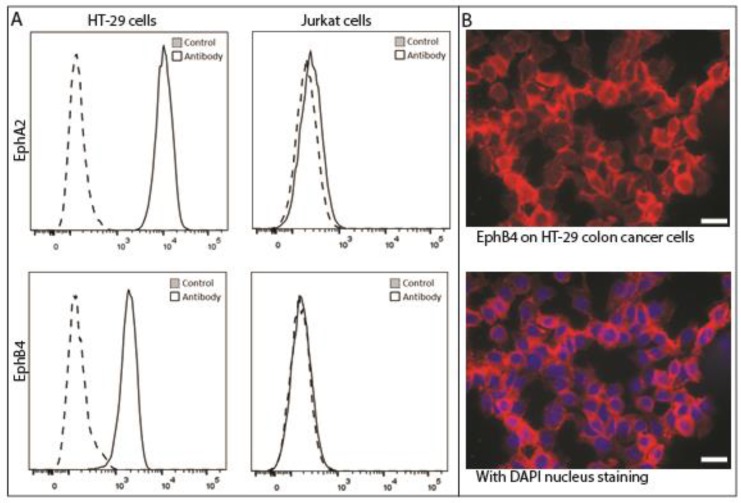
Presence of EphA2 and EphB4 on colon cancer cells. (**A**) Flow cytometry of HT-29 shows abundant presence of both EphA2 and EphB4 compared with negative Jurkat cells; (**B**) Immunofluorescence of EphB4 indicates a membranous staining on HT-29 colon cancer cells grown in monolayer. X-axes indicate fluorescence intensity, the scale bar represents 20 micrometers.

The other most important characteristic for a protein to become a candidate target for imaging next to upregulation in the tumor cells is the availability on the cell membrane of these cells. Being receptors, the majority of the EphA2 and EphB4 would be expected on the cells rather than inside. Figure 1C shows that in our IHC evaluation this is clearly the case for EphB4 and, to a lesser extent for EphA2. Because of the observed advantages of EphB4 with respect to upregulation and cellular localization in IHC, we performed in vitro experiments on various tumor cell lines to evaluate whether EphB4 is targetable on these cells. All cancer cell lines, i.e., lung, breast, and colon, showed an enhanced presence of both EphA2 and EphB4 compared with Jurkat control cells, as shown for colon cancer cell line HT-29 in Figure 3A. A chamber slide assay confirmed the accessibility of EphB4 on HT-29 cells grown on chamber slides in monolayer (Figure 3B).

## 3. Discussion

In theory, any upregulated protein present in cancer tissue could be a potential target for IGOS, but in practice the proteins expressed on the surface of tumor cells are the best candidates [3]. Cheever and colleagues ranked EphA2 as one of the highest cell surface antigens for cancer treatment [4]. Therefore, in this study, we evaluated whether the EphA2 receptor and the related EphB4 receptor are suitable for use in image-guided colorectal cancer surgery [5,6,7,12,13].

Our IHC evaluation of 168 sets of N/T tissue showed a statistical significant upregulation in tumor tissue compared with normal tissue for both EphA2 and EphB4 in the majority of the patients. Both Eph receptors were detected in tumors of various stages and grades and in tumor areas that were suitable for imaging. Therefore, both Eph receptors seem to be valid candidate targets for IGOS of colorectal cancer. However, as clearly illustrated by the patient specific N/T scorings diagram, the value of EphA2 as a clinically usable target is problematic because of the presence of high EphA2 staining in some of the normal tissues. This phenomenon is much less evident for EphB4, making this receptor the better candidate target for IGOS. The higher EphB4 expression in colon cancer compared to normal mucosa was previously also shown at the mRNA level in 62 tissue pairs, with validation at the protein level in only a few samples [14]. Although we did not find a significant correlation between EphA2 or EphB4 expression and the stage or differentiation grade of the tumors, earlier studies found both EphA2 and EphB4 to be preferentially upregulated in the early cancer stages (Stages I and II) [5,6,7,8,9,15]. Over-expression in early colorectal cancer stages is particularly advantageous for imaging during surgery, given the increased difficulty in distinguishing normal and tumor tissue morphologically and the fact that, for early stage tumors, surgery is often not accompanied by chemotherapy. Interestingly, earlier studies using Q-PCR to analyze 53 paired normal and colorectal cancer samples showed that EphB4 is more significantly overexpressed in tumor tissue compared to normal tissue than EphA2 [5,12]. This characteristic supports the choice of EphB4 as a target for IGOS.

Multiple publications have shown that EphA2 overexpression is strongly correlated with tumor progression; consequently, high EphA2 levels are associated with worse patient survival [8,13,16]. High EphB4 expression has also been correlated with poor patient survival [17,18,19], although some studies have also found an opposite correlation [15]. The signaling effects of EphA2 and EphB4 in cancer are complex, and not only tumor-promoting but also tumor-suppressing effects have been reported for these receptors [7,20,21,22]. However, neither a role in carcinogenesis nor the status as a prognostic factor is relevant for the use of a protein as a target for IGOS.

The main disadvantage of our study is the use of a TMA consisting of three cores per tumor, which makes evaluation of the degree of intra-tumor heterogeneity more difficult than with the use of whole tumor sections. Another point of criticism could be the antibodies used for IHC. Although we evaluated a panel of antibodies for EphA2 as well as EpHB4, the evaluation of the distribution, the scoring, and the calculations were performed with the antibody that gave the most consistent results. The selected antibodies were commercially available and both raised against C-terminal peptides of respectively EphA2 and EphB4. In general, antibodies against intracellular domains are well suited for IHC, but in most cases these antibodies are not particularly fit for flow cytometry or for determination by IHC, whether the staining is membranous rather than intracellular. Especially the antibody for EphA2 proved to be less suitable for the latter purpose. Based on these results, we cannot conclude that EphB4 is more suitable as a target for IGOS than EphA2. The flow cytometry data, performed with polyclonal antibodies against whole proteins rather than internal domains, indicated that EphA2 is at least as abundantly present on living cancer cells as EphB4. The incubation of these colon cancer cells with labeled antibodies against EphB4 demonstrates the principle that targeting of this receptor could be clinically relevant, but this should be further elucidated in pre-clinical studies using relevant mouse models.

Targeting of Eph receptors for tumor imaging purposes could be achieved using various agents, such as peptides or antibodies [23,24]. Antibodies offer the advantage of high specificity and affinity, and, thanks to molecular engineering, even bi- and tri-specific antibodies have been developed recently, which could enhance tumor specificity [7,13,25]. Recently, a series of EphB4 antibodies has been evaluated for the use of Positron Emission Tomography (PET) in mice xenografted with human HT-29 colon cancer and MDA-MB-231 breast cells, underscoring the value of EphB4 as a target for tumor imaging [23]. A recent study also developed NIR fluorescence probes for targeted imaging using an EphB4 antibody [26]. While the use of PET for colon cancer is not suitable for routine clinical use [27], NIR fluorescence-based imaging of colorectal cancer shows great potential, especially for rectal cancer [28].

## 4. Materials and Methods

### 4.1. Tissue Micro Array (TMA)

Formalin-fixed paraffin-embedded (FFPE) tissue blocks of primary tumors and their respective normal tissues were collected from the pathology department of the Leiden University Medical Centre (Leiden, The Netherlands). Sections were cut for hematoxylin-eosin staining and histopathologically representative tumor regions were used for preparation of TMA blocks. From each donor block, three 0.6 mm diameter tissue cores were punched from tumor areas and transferred into a recipient paraffin block using a custom-made precision instrument. Because the TMA was designed to evaluate the expression of EphA2 or EphB4 throughout the whole tumor, cores where taken from three different locations across the tumor tissue, plus one outside the tumor, in healthy looking tissue.

### 4.2. Immunohistochemistry

IHC staining of the TMA was performed on 4 μm sections cut from each TMA receiver block. TMA sections were deparaffinized and rehydrated. Endogenous peroxidase was blocked for 20 min in 0.3% hydrogen peroxide in water. The slides were treated for antigen retrieval in citrate buffer (pH 6) for 10 min at 95 °C (DAKO PT Link, Glostrup, Denmark). Sections were incubated overnight with primary antibodies. The antibodies used for EphA2 staining were a polyclonal rabbit (34-7400, Invitrogen/Thermo Fisher Scientific, Waltham, MA, USA) and a polyclonal goat (AF3035, R&D Systems, Minneapolis, MN, USA). Antibodies used for EphB4 were a monoclonal mouse IgG1 (37-1800, Life Technologies/Thermo Fisher Scientific) and a polyclonal goat (AF3038, R&D Systems). The optimal dilution for staining colon cancer tissue sections was optimized for all 4 antibodies. After 30 min of incubation with DAKO envision containing horseradish peroxidase conjugated goat anti-rabbit, goat anti-mouse, or rabbit anti-goat antibodies (DAKO Cytomation, Glostrup, Denmark), the sections were visualized using a diaminobenzidine solution (DAB+; DAKO kit). The sections were counterstained with hematoxylin, dehydrated, and mounted with pertex (Histolab). The entire slides were scanned with a Philips Ultra Fast Scanner 1.6 RA (Philips, Eindhoven, The Netherlands) for further analysis.

### 4.3. Scoring Method

The TMA sections were semi-quantitatively scored for EphA2 or EphB4 staining by two independent examiners (MS, HP) using Philips Digital Pathology Solutions-software (Philips). TMA cores were used when 50% or more was occupied by tissue. The intensity and percentage of positive tumor cells within each core were scored independently and categorized. The intensity score of epithelial staining was defined as 0 when there was no staining; 1 for weak staining; 2 for moderate staining; or 3 for strong staining. The percentage of cells stained was scored 0 when the percentage was 0%; 1 when <25% was stained; 2 for 25%–50% staining; 3 for 50%–90% staining and 4 when >90% of the cells was stained. The two scored parameters were added up into a final score (0–7) for each core. Only tumors with a minimum of 2 cores were used. The median of the score for each tissue was used for data analysis. Only complete sets of tumor and normal tissue were used for further analysis, for a total of 168 tumors.

### 4.4. Cell Culture, Flow Cytometry, and Chamber Slide Assay

Cancer cell lines A549 (lung), BT-20 (breast), HT-29 (colon), and Jurkat (leukemic T-cell lymphoblast) were grown in RPMI or DMEM (Gibco, LifeTechnologies, Carlsbad, CA, USA) as appropriate, with 10% fetal calf serum and 100 IU/mL penicillin/streptomycin (Gibco) at 37 °C in a humidified incubator with 5% CO_2_. The presence of Eph2A and Eph4B on the membranes of these cells was determined by flow cytometry. Cells were cultured until 90% confluence and detached with trypsin/EDTA. Viability of the cells was evaluated with trypan blue. The cells were incubated with 4 μg/mL polyclonal goat antibodies AF3035 and AF3038, against respectively Eph2A and Eph4B for 30 minutes on ice, washed with ice cold phosphate buffered saline pH7.5 (PBS), and incubated with anti-goat secondary antibody conjugated with FITC (A11078, Life Technologies). The cells were then centrifuged, washed, and suspended in PBS containing propidium iodide to exclude dead cells and consequently analyzed in a BD LSRII flow cytometer (BD Biosciences, San Jose, CA, USA) with FlowJo (Tree Star Inc., Ashland, OR, USA). For the plate assay, HT-29 colon cancer cells were grown in 8-well chamber slides (Thermo Scientific, Waltham, MA, USA) under conditions described above. At 75%–80% confluence, the cells were fixated with 4% paraformaldehyde for 10 minutes. After washings with PBS, the cells were incubated with a rabbit monoclonal anti-EphB4 antibody conjugated with phycoerythrine (SinoBiological Inc., LuDong Area, BDA, Beijing, China). The cells were washed and dried. The slides were covered with Prolong Gold with DAPI (Life Technologies) and visualized using a Leica DFC350 FX fluorescence microscope (Leica, Wetzlar, Germany).

### 4.5. Statistical Analysis

Statistical analyses were conducted using SPSS statistical software (version 20.0 for Windows, SPSS Inc., Chicago, IL, USA). Scores are presented as mean ± standard deviation. Differences between groups are calculated using a Student’s paired *t*-test. For the difference in differentiation in relation to tumor stage, a *chi*-square test was used. All statistical tests were conducted two-sided, and *p*-values of 0.05 or less were considered significant.

## 5. Conclusions

In conclusion, both EphA2 and EphB4 show potential as target for image-guided colorectal cancer surgery, but EphB4 seems to have the best characteristics with respect to tumor/normal mucosa distribution, as shown in a relatively large cohort of 168 patients. The in vitro binding data confirm the presence of EphB4 on the cell membrane, which is the other important prerequisite for candidate targets for IGOS.

## Figures and Tables

**Table 1:** Patient and tumor characteristics of the 168 colorectal cancer patients in this study.

Characteristic	Group	Percentage (%)
Gender	Male	48.1
	Female	51.9
Age	<50	11.3
	≥50	88.7
Stage	I	20.3
	IIA	25.0
	IIB	1.9
	IIIA	3.8
	IIIB	21.8
	IIIC	8.3
	IV	18.6
Differentiation	Good	25.7
	Moderate	64.2
	Poor	10.2

## Supplementary Materials

The following are available online at www.mdpi.com/1422-0067/18/2/307/s1.

## Abbreviations

IGOS	Image-guided oncologic surgery
NIH	National Institutes of Health
TMA	Tissue microarray
IHC	Immunohistochemistry
FFPE	Formalin-fixed paraffin-embedded
PET	Positron emission tomography
