# Supplementary material for: Evaluation of EphA2 and EphB4 as Targets for Image-Guided Colorectal Cancer Surgery
Source: Int J Mol Sci. 2017 Feb 3;18(2):307. doi: 10.3390/ijms18020307 (PMC5343843; doi:10.3390/ijms18020307)
Supplement: Supplementary file 1 [file ijms-18-00307-s001.docx]

Supplementary Materials: Evaluation of EphA2 and EphB4 as Targets for Image-Guided Colorectal Cancer Surgery

Marieke A. Stammes, Hendrica A.J.M. Prevoo, Meyke C. Ter Horst, Stéphanie A. Groot, Cornelis J.H. Van de Velde, Alan B. Chan, Lioe-Fee de Geus-Oei, Peter J.K. Kuppen, Alexander L. Vahrmeijer, Elena B. Pasquale and Cornelis F.M. Sier

**Table 1.** IHC scores for both EphA2 and EphB4 in normal and tumoral tissues for each patient.

| **Patients** | **IHC Scores** | | | |
| --- | --- | --- | --- | --- |
|  | **EphA2** | | **EphB4** | |
|  | **Normal** | **Tumor** | **Normal** | **Tumor** |
| 1 | 2 | 3 | 2 | 3 |
| 2 | 0 | 6 | 3 | 0 |
| 3 | 0 | 6 | 4 | 2 |
| 4 | 2 | 6 | 4 | 1 |
| 5 | 0 | 5 | 3 | 4 |
| 6 | 0 | 6 | 3 | 0 |
| 7 | 0 | 6 | 7 | 7 |
| 8 | 6 | 6 | 5 | 5 |
| 9 | 0 | 7 | 6 | 5 |
| 10 | 0 | 5 | 6 | 2 |
| 11 | 0 | 4 | 2 | 5 |
| 12 | 0 | 5 | 4 | 4 |
| 13 | 2 | 5 | 5 | 3 |
| 14 | 2 | 7 | 0 | 6 |
| 15 | 4 | 3 | 6 | 3 |
| 16 | 2 | 4 | 6 | 5 |
| 17 | 0 | 7 | 4 | 2 |
| 18 | 4 | 5 | 0 | 5 |
| 19 | 0 | 4 | 4 | 5 |
| 20 | 3 | 7 | 2 | 3 |
| 21 | 4 | 6 | 2 | 0 |
| 22 | 0 | 5 | 3 | 5 |
| 23 | 2 | 6 | 3 | 5 |
| 24 | 1 | 5 | 3 | 4 |
| 25 | 0 | 6 | 4 | 5 |
| 26 | 2 | 6 | 4 | 0 |
| 27 | 0 | 6 | 3 | 3 |
| 28 | 0 | 5 | 3 | 6 |
| 29 | 0 | 4 | 0 | 7 |
| 30 | 0 | 2 | 6 | 6 |
| 31 | 0 | 4 | 2 | 6 |
| 32 | 2 | 5 | 0 | 4 |
| 33 | 3 | 5 | 4 | 3 |
| 34 | 5 | 6 | 3 | 6 |
| 35 | 0 | 4 | 2 | 2 |
| 36 | 0 | 2 | 3 | 3 |
| 37 | 0 | 4 | 2 | 5 |
| 38 | 3 | 4 | 4 | 5 |
| 39 | 0 | 3 | 3 | 7 |
| 40 | 5 | 6 | 6 | 6 |
| 41 | 1 | 5 | 3 | 5 |
| 42 | 0 | 6 | 3 | 0 |
| 43 | 6 | 6 | 5 | 6 |
| 44 | 2 | 5 | 4 | 5 |
| 45 | 0 | 7 | 4 | 3 |
| 46 | 3 | 4 | 0 | 4 |
| 47 | 2 | 4 | 2 | 6 |
| 48 | 0 | 5 | 2 | 5 |
| 49 | 0 | 7 | 3 | 0 |
| 50 | 3 | 4 | 0 | 0 |
| 51 | 0 | 4 | 6 | 5 |
| 52 | 2 | 6 | 3 | 5 |
| 53 | 6 | 6 | 2 | 7 |
| 54 | 5 | 6 | 6 | 5 |
| 55 | 3 | 7 | 3 | 6 |
| 56 | 6 | 2 | 3 | 6 |
| 57 | 2 | 4 | 3 | 5 |
| 58 | 7 | 6 | 2 | 5 |
| 59 | 4 | 6 | 3 | 5 |
| 60 | 4 | 6 | 3 | 6 |
| 61 | 0 | 0 | 2 | 6 |
| 62 | 5 | 3 | 7 | 5 |
| 63 | 2 | 5 | 3 | 5 |
| 64 | 6 | 5 | 2 | 4 |
| 65 | 2 | 4 | 2 | 6 |
| 66 | 0 | 6 | 0 | 5 |
| 67 | 3 | 4 | 4 | 6 |
| 68 | 2 | 3 | 3 | 6 |
| 69 | 0 | 2 | 4 | 6 |
| 70 | 2 | 2 | 2 | 5 |
| 71 | 2 | 4 | 0 | 3 |
| 72 | 2 | 2 | 5 | 5 |
| 73 | 7 | 4 | 6 | 6 |
| 74 | 4 | 4 | 6 | 5 |
| 75 | 0 | 3 | 4 | 3 |
| 76 | 3 | 6 | 4 | 0 |
| 77 | 4 | 7 | 2 | 5 |
| 78 | 2 | 5 | 3 | 6 |
| 79 | 4 | 4 | 0 | 6 |
| 80 | 7 | 2 | 3 | 6 |
| 81 | 0 | 4 | 2 | 6 |
| 82 | 3 | 3 | 0 | 6 |
| 83 | 6 | 0 | 4 | 5 |
| 84 | 2 | 4 | 4 | 5 |
| 85 | 4 | 7 | 3 | 5 |
| 86 | 0 | 2 | 3 | 5 |
| 87 | 3 | 0 | 3 | 6 |
| 88 | 4 | 0 | 5 | 7 |
| 89 | 3 | 4 | 5 | 5 |
| 90 | 6 | 0 | 3 | 5 |
| 91 | 0 | 0 | 3 | 6 |
| 92 | 2 | 3 | 6 | 5 |
| 93 | 7 | 2 | 3 | 6 |
| 94 | 5 | 3 | 2 | 6 |
| 95 | 2 | 2 | 5 | 5 |
| 96 | 2 | 3 | 3 | 6 |
| 97 | 3 | 1 | 0 | 3 |
| 98 | 5 | 2 | 0 | 6 |
| 99 | 5 | 3 | 0 | 7 |
| 100 | 0 | 0 | 2 | 4 |
| 101 | 6 | 2 | 0 | 5 |
| 102 | 4 | 6 | 0 | 2 |
| 103 | 7 | 7 | 0 | 4 |
| 104 | 2 | 6 | 3 | 6 |
| 105 | 6 | 4 | 2 | 6 |
| 106 | 5 | 2 | 0 | 2 |
| 107 | 6 | 3 | 0 | 3 |
| 108 | 3 | 7 | 0 | 3 |
| 109 | 3 | 5 | 0 | 2 |
| 110 | 0 | 3 | 0 | 6 |
| 111 | 3 | 4 | 3 | 5 |
| 112 | 3 | 2 | 0 | 2 |
| 113 | 4 | 4 | 0 | 4 |
| 114 | 7 | 5 | 0 | 5 |
| 115 | 3 | 0 | 0 | 5 |
| 116 | 2 | 3 | 0 | 4 |
| 117 | 6 | 4 | 2 | 5 |
| 118 | 4 | 0 | 0 | 6 |
| 119 | 7 | 1 | 0 | 6 |
| 120 | 6 | 0 | 0 | 6 |
| 121 | 4 | 5 | 3 | 6 |
| 122 | 3 | 4 | 0 | 5 |
| 123 | 2 | 5 | 0 | 5 |
| 124 | 5 | 7 | 2 | 6 |
| 125 | 4 | 6 | 0 | 6 |
| 126 | 3 | 6 | 3 | 5 |
| 127 | 6 | 5 | 2 | 6 |
| 128 | 6 | 5 | 5 | 5 |
| 129 | 7 | 0 | 0 | 6 |
| 130 | 5 | 6 | 0 | 6 |
| 131 | 4 | 5 | 0 | 6 |
| 132 | 6 | 4 | 0 | 6 |
| 133 | 5 | 4 | 0 | 3 |
| 134 | 5 | 7 | 0 | 5 |
| 135 | 6 | 5 | 3 | 5 |
| 136 | 5 | 4 | 0 | 6 |
| 137 | 7 | 5 | 4 | 5 |
| 138 | 4 | 6 | 0 | 2 |
| 139 | 4 | 6 | 2 | 6 |
| 140 | 4 | 6 | 0 | 7 |
| 141 | 6 | 5 | 3 | 5 |
| 142 | 5 | 6 | 0 | 4 |
| 143 | 6 | 5 | 2 | 6 |
| 144 | 7 | 6 | 4 | 5 |
| 145 | 7 | 6 | 0 | 4 |
| 146 | 6 | 7 | 2 | 5 |
| 147 | 6 | 5 | 2 | 4 |
| 148 | 5 | 5 | 0 | 6 |
| 149 | 6 | 5 | 0 | 5 |
| 150 | 7 | 6 | 0 | 4 |
| 151 | 7 | 4 | 0 | 5 |
| 152 | 6 | 6 | 3 | 5 |
| 153 | 4 | 4 | 0 | 6 |
| 154 | 4 | 5 | 4 | 5 |
| 155 | 3 | 4 | 0 | 2 |
| 156 | 4 | 4 | 2 | 6 |
| 157 | 4 | 6 | 0 | 7 |
| 158 | 4 | 6 | 3 | 5 |
| 159 | 7 | 6 | 0 | 4 |
| 160 | 4 | 4 | 2 | 6 |
| 161 | 4 | 5 | 4 | 5 |
| 162 | 4 | 6 | 0 | 4 |
| 163 | 5 | 6 | 2 | 5 |
| 164 | 4 | 6 | 2 | 4 |
| 165 | 5 | 6 | 0 | 6 |
| 166 | 6 | 6 | 0 | 5 |
| 167 | 7 | 6 | 0 | 4 |
| 168 | 5 | 4 | 0 | 5 |
